# Integrin α4 Enhances Metastasis and May Be Associated with Poor Prognosis in MYCN^low^ Neuroblastoma

**DOI:** 10.1371/journal.pone.0120815

**Published:** 2015-05-14

**Authors:** Shanique A. Young, Katelyn E. McCabe, Alena Bartakova, Joe Delaney, Donald P. Pizzo, Robert O. Newbury, Judith A. Varner, David D. Schlaepfer, Dwayne G. Stupack

**Affiliations:** 1 Division of Gynecologic Oncology, Department of Reproductive Medicine, UCSD School of Medicine, 9500 Gilman Drive, La Jolla, California, 92093, United States of America; 2 University of California San Diego Moores Cancer Center, 3855 Health Sciences Drive, La Jolla, California, 92093, United States of America; 3 University of California San Diego Center for Advanced Laboratory Medicine, 10300 Campus Point Drive, MC7210, Room 1253, San Diego, CA, 92121, United States of America; 4 Department of Pathology, UCSD School of Medicine, 9500 Gilman Drive, La Jolla, California, 92093, United States of America; Seoul National University, KOREA, REPUBLIC OF

## Abstract

High-risk neuroblastoma is associated with an overall survival rate of 30–50%. Neuroblastoma-expressed cell adhesion receptors of the integrin family impact cell adhesion, migration, proliferation and survival. Integrin α4 is essential for neural crest cell motility during development, is highly expressed on leukocytes, and is critical for transendothelial migration. Thus, cancer cells that express this receptor may exhibit increased metastatic potential. We show that α4 expression in human and murine neuroblastoma cell lines selectively enhances *in vitro* interaction with the alternatively spliced connecting segment 1 of fibronectin, as well as vascular cell adhesion molecule-1 and increases migration. Integrin α4 expression enhanced experimental metastasis in a syngeneic tumor model, reconstituting a pattern of organ involvement similar to that seen in patients. Accordingly, antagonism of integrin α4 blocked metastasis, suggesting adhesive function of the integrin is required. However, adhesive function was not sufficient, as mutants of integrin α4 that conserved the matrix-adhesive and promigratory function *in vitro* were compromised in their metastatic capacity *in vivo*. Clinically, integrin α4 is more frequently expressed in non-MYNC amplified tumors, and is selectively associated with poor prognosis in this subset of disease. These results reveal an unexpected role for integrin α4 in neuroblastoma dissemination and identify α4 as a potential prognostic indicator and therapeutic target.

## Introduction

Neuroblastoma (NB) is a highly metastatic childhood cancer that accounts for up to 15% of deaths among pediatric cancer patients [1]. This malignancy most commonly develops sporadically in the adrenal medulla from neuroectodermal cells of neural crest cell origin and spreads to the bone marrow/bone, liver, and lymph nodes [2]. Approximately 70% of NB patients present with disseminated disease at the time of diagnosis [2,3]. NB patients with aggressive disease have an overall survival rate of 30–50%. Amplification of the *MYCN* oncogene is perhaps the strongest indicator of an aggressive tumor. Subsequent identification of pathological markers in *MYCN* amplified disease does not frequently increase prognositic accuracy. However, for tumors without *MYCN* amplification, including stage IV disease in children older than 18 months, markers are poor [4–6]. Thus, identification of key players in NB could aid in more effectively managing this disease.

Metastasis is an extremely complex multi-step process that requires tumor cells to invade local tissues, access and survive in circulation, exit the circulation at a distant site, and adapt and grow in a new tissue microenvironment. This requires orchestrated actions of a variety of cell surface molecules. The integrin family of cell surface adhesion receptors is involved in each step of this process and is an important determinant of metastatic ability [7]. Integrins facilitate binding to the extracellular matrix and other cell surface proteins and translate mechanical and chemical cues into intracellular signals that regulate cell behavior. To date, 18 α and 8 β subunits have been identified and are expressed as 24 unique α-β heterodimers [8]. Each heterodimer can have a distinct impact on cancer progression that is dependent upon a variety of cellular, molecular, and microenvironment-related factors [7].

Among these, integrin α4 is a 150 kilodalton subunit of the α4β1 and α4β7 heterodimers. Integrin α4 facilitates the extravasation of leukocytes from circulation into surrounding tissues via binding to its endothelial ligand, vascular cell adhesion molecule-1 (VCAM-1) [9,10]. Integrin α4 also binds surface-exposed alternatively spliced forms of fibronectin (CS1). During development, α4 is important for neural crest cell motility [11,12]. In transformed neural crest-derived cells, α4 expression may enhance NB adhesion to endothelium, leading to transendothelial migration and increasing NB metastatic potential. In addition, α4 expression promotes leukocyte proliferation and survival and may function similarly in other cell types such as NB [13,14].

The role of integrin α4 in cancer is controversial and is dependent on expression levels, cellular context, and stage of tumor progression [15]. However, we previously provided evidence to suggest that integrin α4 plays a role in promoting malignant behaviours in *MYCN*-amplified neuroblastoma cells. Cells expressing α4 demonstrate enhanced cell motility on select substrates *in vitro*, and α4 expression was associated with increased tumor stage in a small sample of clinical cases [16]. Thus, the role of α4 in NB metastasis is implied but has not been validated *in vivo*.

Our studies here show that integrin α4 is expressed more frequently in primary tumor samples from patients lacking MYCN amplification. In contrast to patients with MYCN amplification, where MYCN is a dominant phenotypic marker, α4 expression may be associated with poor prognosis in these nonamplified patients. In addition, we show enhanced experimental NB metastasis among α4-expressing NB, reconstituting a pattern of organ involvement similar to that seen in patients. This metastasis is dependent upon the presence of the α4 cytoplasmic tail, but require more than simple adhesive and migratory functions, which can be mimicked with chimeric cytosolic domain. These results implicate integrin α4 as a potential target for the control of NB metastasis. Clinical approaches to target α4 in inflammatory diseases might therefore be considered for this aggressive malignancy [17–19].

## Materials and Methods

### Ethics statement

The Institutional Animal Care and Use Committee of the University of California San Diego (UCSD) has approved all animal studies (Protocol #S05356). Written informed consent was obtained from donors, and use of the tissue was approved under the auspices of the UCSD Institutional Review Board (Protocol 071729).

### Antibodies and reagents

Mouse anti-human α4 (P1H4), anti-β1 (P4C10), and anti-β5 (P1F6) antibodies were purchased from Millipore. The production of the GST-CS1 fibronectin has been previously described [20]. Unless otherwise noted, all other reagents were from Sigma.

### Human tissues and cell lines

De-identified human neuroblastoma primary tumor samples were obtained under IRB approval (protocol 071729) from Rady Children’s Hospital, San Diego, California, USA. C1300 cells were provided by Dr. RA Reisfeld [4]. NB5 cells were obtained from Dr, Jill Lahti, St. Jude Children’s Research Hospital [22]. Human NB5 and murine C1300 cells were cultured in RPMI 1640 (Life Technologies) supplemented with 10% fetal bovine serum (FBS) and 1% MEM non-essential amino acids, 1mM sodium pyruvate, and 1% antibiotic-antimycotic solution from Mediatech. HEK-293T cells were cultured in Hi-Glucose DMEM (Mediatech) and supplemented with 10% FBS and 1% MEM non-essential amino acids. All cell lines were grown in a 37°C incubator containing 5% CO_2_ and a humidified atmosphere. Cells were tested regularly for the presence of mycoplasma via PCR. No data from mycoplasma-positive cells was used in these studies.

### Integrin α4 constructs, lentivirus production and establishment of stably expressing cell lines

Enhanced green fluorescent protein (eGFP), α4-GFP, and α4 Δcyto-GFP [23] were shuttled into pCDH lentiviral constructs. The chimeric α4-GFP fusion construct contains a human extracellular domain and a mouse intracellular domain. Constructs were verified by DNA sequencing. Integrin α4 constructs lacking the GFP fusion were developed by inserting (via QuikChange PCR) a stop codon immediately after the termination of the α4 sequence.

For lentivirus production, HEK-293T cells were transfected with a pCDH1 eGFP or an integrin α4 construct and CMV-VSVG envelope vector, RSV-Rev, and pMDL g/p RRE using Lipofectamine 2000 (Invitrogen). Lentiviral particles were harvested 48 hours after transfection. Target cells were transduced in the presence of 8 μg/ml polybrene (Sigma). Expression of GFP, or integrin α4 constructs was verified via flow cytometry.

### Flow cytometry

To examine or isolate cells stably expressing integrin α4 constructs, cells were placed in blocking buffer (2% BSA-PBS) for 15–30 min followed by incubation with 5 ug/ml anti-α4 (P1H4) or primary antibody for 30–45 min on ice. Cells were washed with blocking buffer and incubated with 1 ug/ml secondary antibody (Alexa Fluor 647, Invitrogen) for 30 min on ice. Cells were washed, resuspended in PBS and analyzed/sorted for α4 and GFP expression.

### Cell proliferation and soft agar growth

To measure cell proliferation, cells were seeded in a 6-well tissue culture-treated plate and incubated at 37°C for up to 6 days. At various time points, cell number and viability (trypan blue exclusion) was assessed using the Vi-Cell XR (Beckman Coulter). For soft agar assays, 48-well dishes were coated with a 0.5% bottom layer of Difco Agar Noble (BD Biosciences) in complete RPMI and allowed to solidify. Cells were then seeded in 0.3% agar-RPMI. Once solidified, complete RPMI was added to the top of each well. Growth was monitored daily. For quantification, colonies were stained with 0.05% crystal violet in methanol, imaged and counted.

### Cell adhesion and migration

For cell adhesion, 48-well non-tissue culture treated plates were coated with 150 μL of PBS or substrate overnight at 4°C. Plates were blocked with 2.5% BSA for 1 hour at 37°C. Cells were trypsinized and treated with a 1:1 ratio of trypsin neutralizing solution (Lonza) and resuspended in adhesion medium (serum-free DMEM). Cells were held in suspension for 30 minutes in a 37°C water bath. 1 x 10^5^ cells in 200 μL were seeded per well in triplicate and allowed to adhere for 30 minutes at 37°C. Wells were washed gently with adhesion medium to remove unattached cells. Cells were then stained for 10 minutes with 0.1% crystal violet in methanol and washed with water. Plates were allowed to dry. The crystal violet was then reabsorbed with 100 μL of methanol and transferred to a 96-well plate for analysis. Absorbance was measured at 600nm using a spectrophotometer (BioTek).

For haptotaxis cell migration assays, cells were starved for 16–18 hours in 0.5% FBS in DMEM. Following trypsinization and quenching with TNS, 1 x 10^5^ cells in 300 μL of migration medium (0.5% BSA-DMEM) were seeded into transwells (6.5mm diameter, 8um pore size; Fisher Scientific) coated on the bottom side of the membrane with substrate. Cells were pre-incubated with 10 ug/ml antibody where indicated. Cells were allowed to migrate for 3hrs at 37°C. Cells were then fixed with 0.1% crystal violet in methanol and all cells on the upper side of the filter were removed. Migration was quantified via spectrometry of resolubilized crystal violet or by direct counting of cells using light microscopy.

For wound healing studies, 24-well non-TC plates were coated with 300 μL of the desired substrate over night at 4°C. Plates were blocked for 1 hr in 2.5% BSA then cells were seeded at confluence for 3hrs in 10% FBS complete RPMI. A wound was created in each well using a 10 μL pipette tip (BioPioneer GSO76508). In order to standardize wound width, tips were preconditioned by scratching the inner lid of the 24-well non-TC plates ten times. Wells were washed and cells were allowed to migrate in 1% FBS RPMI in a heated chamber supplemented with CO_2_. Timelapse microscopy on the Olympus IX51 was used to capture migration every 20 minutes for 18 hours. Migration was quantified by analyzing the pixel area of the initial and final wound (Adobe Photoshop).

### Animal studies

Syngeneic A/J mice (Jackson Labs) were used for C1300 *in vivo* studies. For tail vein injections, 1x10^6^ cells were injected into 8-week-old mice in 100 μL saline. Cells were pre-incubated in anti-α4 (P1H4) or IgG control antibody where indicated. Mice were sacrificed after 18–24 days and examined for metastatic lesions. Macroscopic liver lesions were counted after Bouin’s staining. For subcutaneous studies, 0.5–1x10^6^ cells were injected into the flank region in 50–100 μL saline. Tumors were harvested after 10–14 days. For adrenal gland injections, mice were anesthetized with isofluorane (Med-Vet International). Buprenorphine was administered as a pre- and post-operative analgesic. The surgical area was depilated and disinfected. 2 x 10^5^ cells in 2 μL saline were injected directly into the adrenal gland through a small suprarenal incision. The peritoneum was sutured closed and the skin was closed with wound clips. Mice were monitored for 2 weeks then euthanized and examined for tumors.

### Immunohistochemistry (IHC)

For IHC on 28 unique human primary neuroblastoma samples, tissue sections were cut from blocks of formalin-fixed paraffin-embedded tumor tissue. Four-micron tissue sections were stained with rabbit anti-integrin α4 (1:150; Cell Signaling, #8440) or mouse anti-CD45 (1:250; Cell Marque, LCA 145M-94). Antigen retrieval was performed using Cell Conditioning 1 (CC1) for 48 minutes at 95°C. Primary antibody was visualized using DAB as a chromagen using the UltraMap system (Ventana Medical systems) followed by hematoxylin as a counterstain. Slides were rinsed, dehydrated through alcohol and xylene and coverslipped.

For IHC on tissues from animal studies, sections from tissues frozen in OCT medium were fixed in ice-cold acetone for 10 minutes, rehydrated for 5 minutes in wash buffer (0.5% BSA-PBS) and blocked for 15–30 minutes in 1.25% normal goat serum (NGS) in PBS. Tissues were washed and incubated with a mouse anti-human α4 antibody (1:200; Millipore, P1H4) overnight followed by washing and incubation with goat anti-mouse AlexaFluor 647 (Invitrogen, A21235), a fluorophore-conjugated secondary antibody, for 30 minutes. (Note: C1300 cells stably express the chimeric integrin α4 construct, which contains a human extracellular domain.) Following the final washes, tissues were coverslipped using Dako Fluorescent Mounting Medium. Hematoxylin and eosin (H&E) staining was performed by the UCSD Moores Cancer Center Histology & Immunohistochemistry Core. For H&E sections, tissues were fixed in 10% zinc formalin (Fisher). Following deparaffinization with xylene and rehydration, tissues were stained with Gill II Hematoxylin and eosin (Surgipath). Slides were then rinsed, dehydrated through alcohol and Citrisolv (Fisher) and coverslipped.

### Database analyses

Expression array data were evaluated using the R2: Genomics analysis and visualization platform (http://r2.amc.nl) developed within the Department of Oncogenomics of the Academic Medical Center (AMC) in Amsterdam, the Netherlands. The Seeger dataset used included gene expression and patient relapse data. The ITGA4 (213416_at) probe was used. The query parameter was relapse-free survival. For the Kaplan-Meier analysis of the Seeger dataset, the cut-off value was 38.2. Raw and Bonferroni p values were calculated via the website interface.

### Statistical analysis

Unless otherwise noted, values for adhesion, haptotaxis, and proliferation are means ± standard deviation (s.d.) from a representative experiment performed at least three times in triplicate. Values for soft agar growth assays are means ± s.d. from a representative experiment performed at least two times with 6 replicates each. For *in vitro* studies, where absolute values ranged, the study yielding the median result is shown as representative. For all experiments, the differences between groups were assessed via a two-tailed Student’s t-test or ANOVA followed by the Tukey test for *in vitro* assays and via the Wilcoxon Rank Sum Test/Mann Whitney U test for *in vivo* assays. A P<0.05 was set as the level of statistical significance.

## Results

### Integrin α4 may be associated with poor prognosis in non-MYCN amplified patients

**Fig 1:**
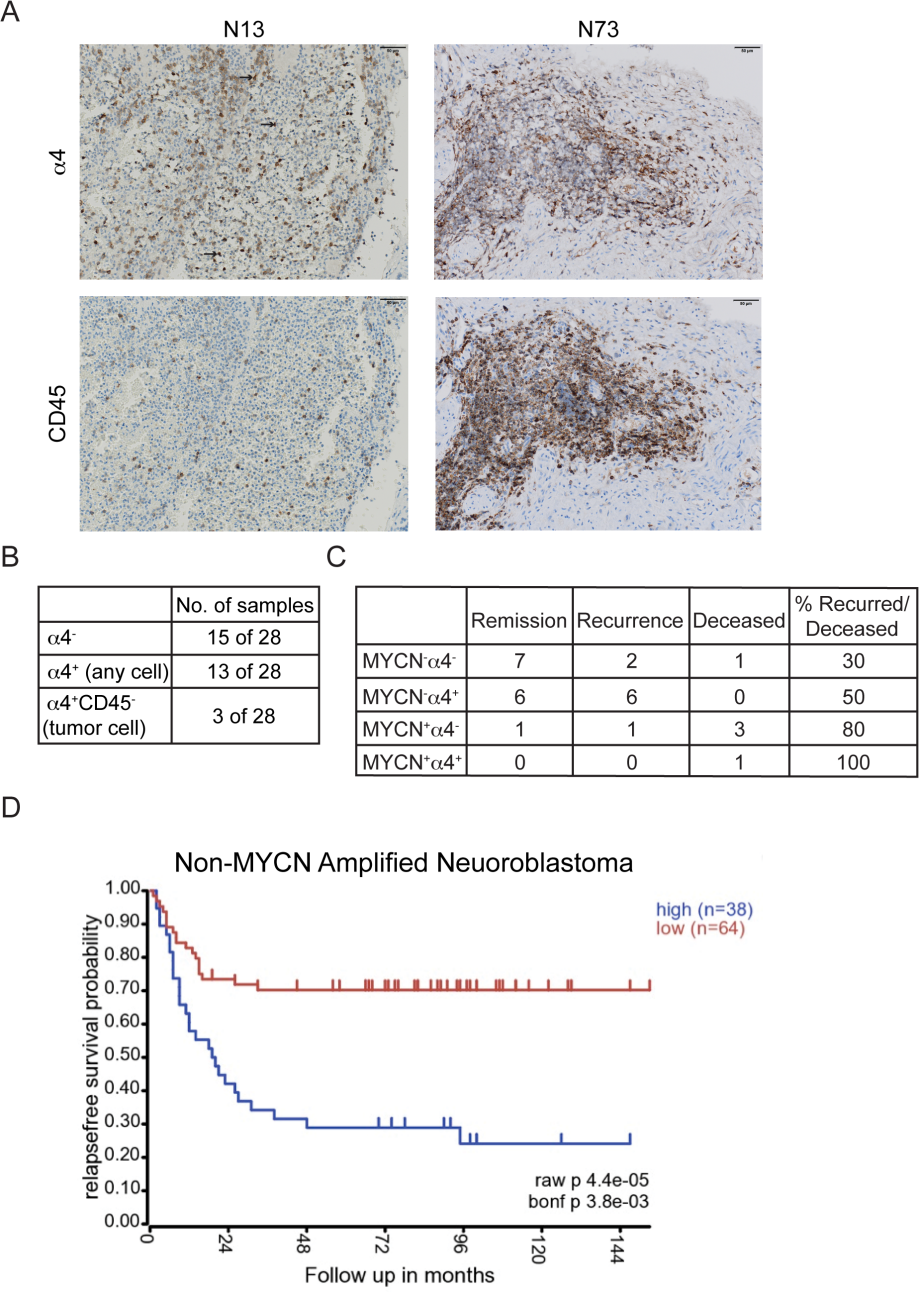
Integrin α4 expression may be associated with worse prognosis in MYCN^low^ NB. Twenty-eight human primary neuroblastoma samples were stained for integrin α4 (HRP). Serial sections of α4 positive samples were stained for CD45. (A) Example of NB patient samples with tumor cell and leukocyte α4 staining (N13) or leukocyte α4 only (N73) Black arrows indicate α4-positive tumor cells (CD45^**-**^). (B) Integrin α4 status of human NB samples. (C) For the 28 samples analyzed, patient status was stratified by MYCN amplification and integrin α4 expression then grouped according to their last known disease status. (D) Kaplan-Meier curve of relapse-free survival for non-MYCN amplified patients with high or low α4 gene expression (raw p = 4.4 x 10^**–5**^; bonf. p = 3.8 x 10^**–3**^). Analysis was performed using the Seeger dataset from the R2: Genomic analysis and visualization platform.

We previously demonstrated that cultured neuroblastoma cell lines quite commonly express integrin α4, which can contribute to signaling via non-receptor tyrosine kinases following ligation of fibronectin in MYCN-amplified tumor cell lines, such as NB8 [16]. To begin to evaluate the incidence of integrin α4 expression in tumors, we analyzed a panel of 28 clinical samples by immunohistochemistry. Integrin α4 expression in tissues was heterogeneous (Fig 1A), and the incidence of positive cells was somewhat lower in patients than in cell lines. Serial sections from positive samples were co-stained with anti-CD45 to detect inflammatory cells and to more clearly assign positivity to tumor cells. Integrin α4 expression was observed on nearly 50% of tumors. Among these, however, inflammatory cell expression was the most commonly observed phenomenon; nonetheless, there was putative tumor cell (CD45-, CD49d+) expression in 3 of the 28 cases (Fig 1B).

Interestingly, α4 was expressed more frequently in samples from patients lacking MYCN amplification (12/22 vs. 1/6 of α4-positive samples) (Fig 1C). In general, the presence of α4 expression on any cell type in the tumor correlated with worse prognosis in the MYCN^low^ tumors (3/10 vs 6/12 recurred/deceased). To extend these results, we analyzed α4 gene expression among non-MYCN amplified patients. Kaplan Meier analysis revealed a significantly decreased probability of relapse-free survival among patients expressing high levels of integrin α4 (Fig 1D). MYCN status alone is sufficient to predict poor outcome in 20–25% of primary neuroblastoma [1,24]. However, those tumors lacking amplification of this oncogene are usually classified into low or intermediate risk groups [25]. Integrin α4 may serve as an additional prognostic marker to further delineate therapeutic regimens for these patients.

### Integrin α4 promotes motility of neuroblastoma cells

The negative prognostic value of α4 integrin expression in microarray analysis (Fig 1D) may indicate a robust inflammatory component rather than the tumor component. Moreover, since dorsal root ganglion neurons transiently express integrin α4 during outgrowth [26], the capacity to express integrin α4 on some or all cells in NB tumors may simply indicate tumor plasticity. Thus, it was not immediately clear whether tumoral integrin a4 would provide any metastatic advantage.

**Fig 2:**
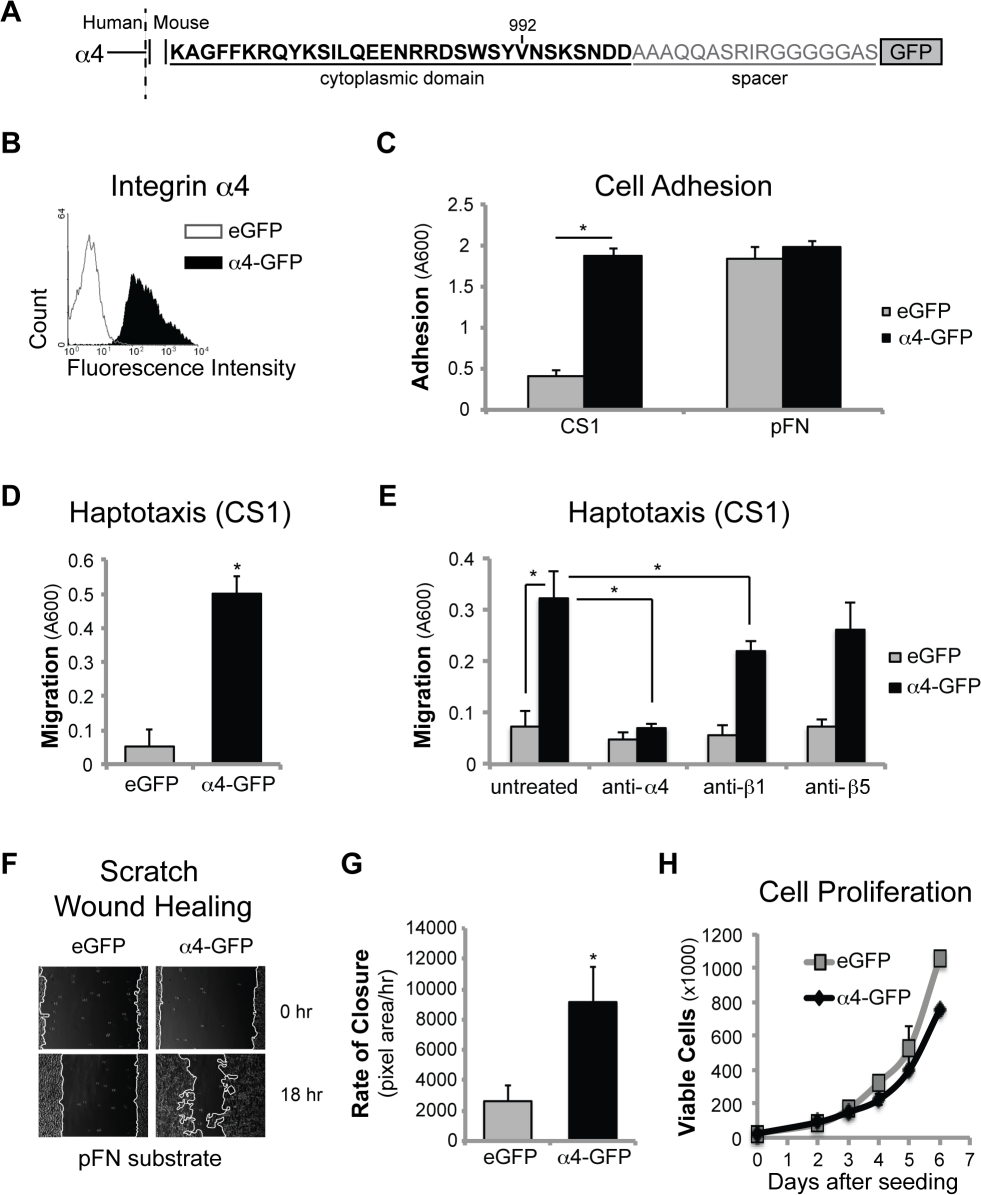
Integrin α4 promotes human NB cell adhesion and migration. (A) Map of the cytoplasmic domain of the chimeric integrin α4-gfp fusion protein. (B) Flow cytometry analysis of NB5 cells stably expressing a full-length integrin α4-GFP fusion construct (shaded peak) or an eGFP control vector (open peak) (α4 antibody; P1H4). (C) Attachment to 5 ug/ml GST-CS1 FN (p<0.00001) or plasma FN (30 minutes). (D) Transwell haptotaxis to 5 ug/ml GST-CS1 FN for 3 hours (p<0.001). (E) Haptotaxis to GST-CS1 FN of C1300 eGFP and α4-GFP cells pre-treated with 10 ug/ml of antibodies against integrins α4 (P1H4) (p<0.01), β1 (P4C10) (p<0.05) or β5 (P1F6). (F) Scratch wound healing on 5 ug/ml pFN. (G) Quantification of wound closure in E (p<0.001). (H) Proliferation (by live cell count) over 6 days. Values for wound healing migration are means ± s.d. (n = 6) for a representative experiment (of three).

Therefore, to directly test whether neuroblastoma with low expression of MYCN could derive a biological advantage from α4 integrin expression, we established stable cell lines ectopically expressing integrin α4 with a c-terminal GFP-tag in the MYCN^low^ (NB5) NB cell line [27–29] (Fig 2A). Parental NB5 cells lack this integrin. Integrin α4 expression was detected via flow cytometry (Fig 2B). An increase in integrin β1 expression was observed concordant with α4-gfp expression, but no other significant changes associated with α4 reconstitution were observed (S1 Fig).

We next undertook functional assays, including adhesion and haptotaxis toward an α4-specific ligand, the connecting segment 1 (CS1) of fibronectin, and to plasma fibronectin (pFN), a ligand that includes both CS1 and arginine-glycine-aspartic acid (RGD) sites bound by other integrins (Fig 2C and 2D). Integrin α4 expression enhanced adhesion on, and haptotaxis toward, the CS1 domain. There was no difference in adhesion to pFN, consistent with the expression of other fibronectin-binding integrins on these cells (S1 Fig).

To evaluate whether the increased migration was due to the expression of integrin α4, we tested whether antagonism of integrin α4 impacted the ability of α4 to promote haptotaxis. Antibodies to integrin α4 significantly reduced migration of the NB5-α4 cells, while antagonism with antibodies against a non-CS1 binding integrin had no significant effect on cell migration in the modified Boyden chamber assay (Fig 2E).

Haptotaxis is tightly associated with adhesion, and may not reflect the dynamic turnover of complexes necessary for sustained cell migration. Therefore, we also evaluated the effect of α4 expression in a two-dimensional wound closure assay. Cells expressing α4 had significantly faster wound closure on fibronectin (Fig 2F and 2G) suggesting that the effects of integrin a4 were extended beyond haptotaxis to sustained migration as well. Importantly, α4 integrin expression had no significant impact on 2D cell proliferation (Fig 2H), suggesting that proliferation during the migration interval was insufficient to explain the rapidity of would closure.

Similar results were obtained in a MYCN-amplified tumor cell line (NB8) with endogenous α4 expression (S2 Fig). Adhesion and migration on CS1 were significantly reduced in a NB8 subline selected for the α4 negative population (SAN) [16] and reconstituted with a GFP control vector (SAN+GFP). However, reconstitution with integrin α4 (SAN+α4-GFP) rescued α4-mediated adhesion and migration, as we have previously shown (16). Together, these results support the concept that integrin a4 expression in human NB cells promotes migration.

### Integrin α4 expression does not impact primary tumor growth

**Fig 3:**
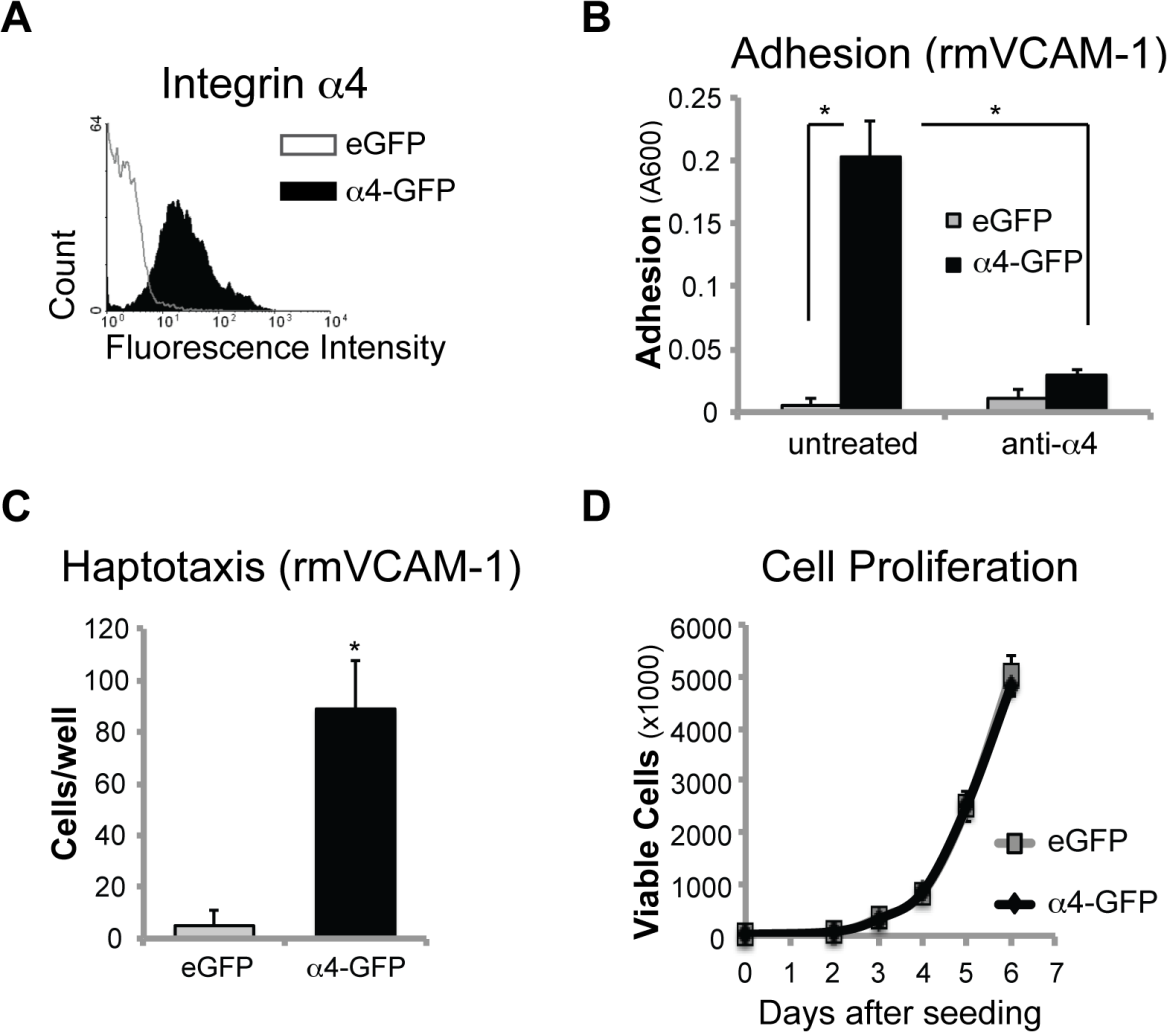
Integrin α4 promotes mouse NB cell adhesion and migration. (A) Flow cytometry analysis of C1300 cells stably expressing a full-length integrin α4-GFP fusion construct (shaded peak) or an eGFP control vector (open peak) (α4 antibody; P1H4). (B) Adhesion of cells that were untreated (p<0.001) or pre-treated with an anti-α4 antibody (P1H4) to 2 ug/ml recombinant mouse (rm) VCAM-1 after 30 minutes (p<0.001). (C) Haptotaxis to 2 ug/ml rm-VCAM-1 after 3 hours (p<0.01). (D) Proliferation (by live cell count) over 6 days.

To extend these studies to an *in vivo* mouse model, we established a new cell line that stably expressed the α4-GFP fusion protein (Fig 3A) in C1300 mouse neuroblastoma that lacked endogenous expression of integrin α4 and lack MYCN amplification [30]. Mirroring the results in the human cells, α4 expression promoted adhesion and haptotatic migration of C1300 cells towards the α4 ligand, VCAM-1 (Fig 3B and 3C). As in the human system, α4 had no effect on proliferation (Fig 3D).

**Fig 4:**
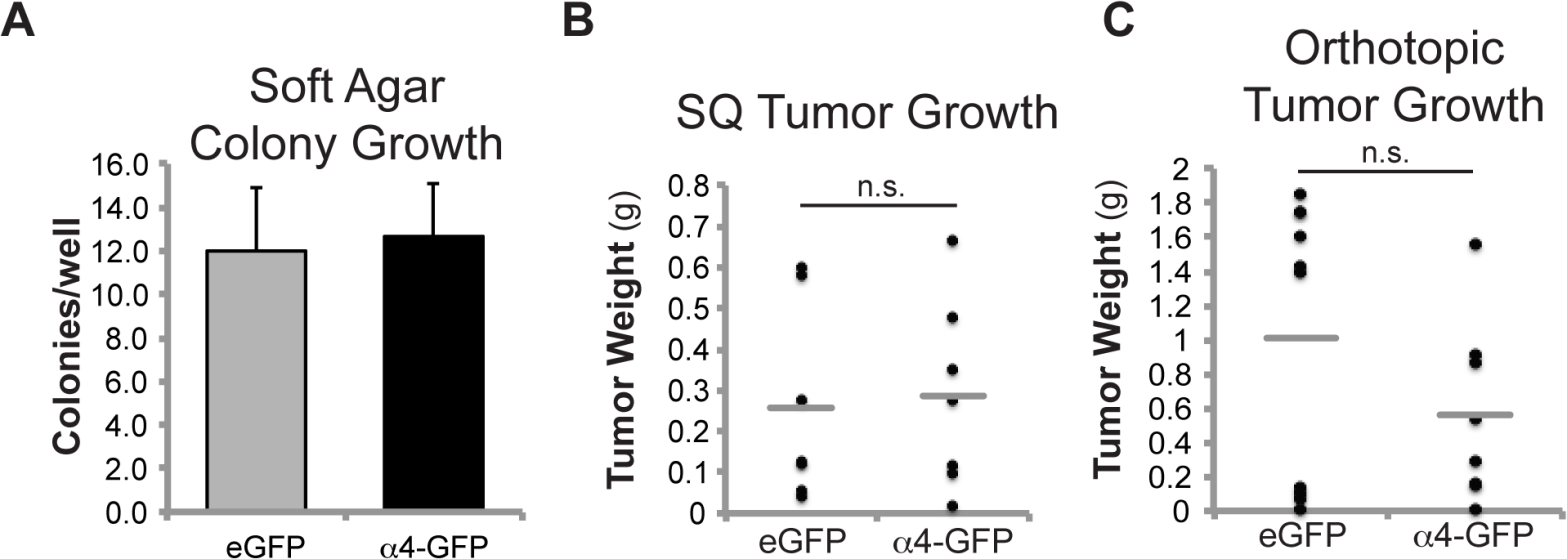
Integrin α4 does not drive NB tumor growth. (A) Soft agar colony growth of C1300 eGFP or α4-gfp cells after 4 days. (B) C1300 eGFP or α4-GFP cells were injected subcutaneously (SQ) into 8-week-old A/J mice (n = 7). Tumors were harvested and weighed 11 days later. Each filled circle represents one mouse. Gray lines denote the means of each group. (C) C1300 eGFP or α4-GFP cells were injected into the adrenal gland (orthotopic site) of 8-week-old A/J mice (n = 10 eGFP, 8 α4-GFP). After 2 weeks, tissues were harvested and examined for macroscopic tumors.

Although integrin α4 did not impact cell proliferation *in vitro*, it remained unclear whether it would influence primary NB tumor growth *in vivo*. To evaluate this, we first performed soft agar colony growth (Fig 4A), and found no difference in C1300 cell survival associated with integrin α4. Then, we injected C1300 cells subcutaneously into the flank of syngeneic A/J mice (Fig 4B). In this case, we observed no significant differences in tumor growth between the α4-GFP and eGFP controls.

Since the principal ECM component in skin is highly enriched for collagen, a non-ligand for integrin α4, and since we previously documented the negative impact of unligated integrins on NB survival, the subcutaneous model might not reflect the potential advantage offered by expression of the α4 receptor. The most common site of origin for NB tumors is the adrenal gland [1]. Therefore, control NB or NB cells expressing α4 were seeded into the adrenal gland of A/J mice and orthotopic tumors allowed to develop over two weeks. Perhaps surprisingly, orthotopic tumor growth was observed to be consistent with subcutaneous growth (Fig 4C), validating the observation that integrin α4 expression had no significant impact on primary tumor growth.

### Integrin α4 promotes neuroblastoma metastasis

**Fig 5:**
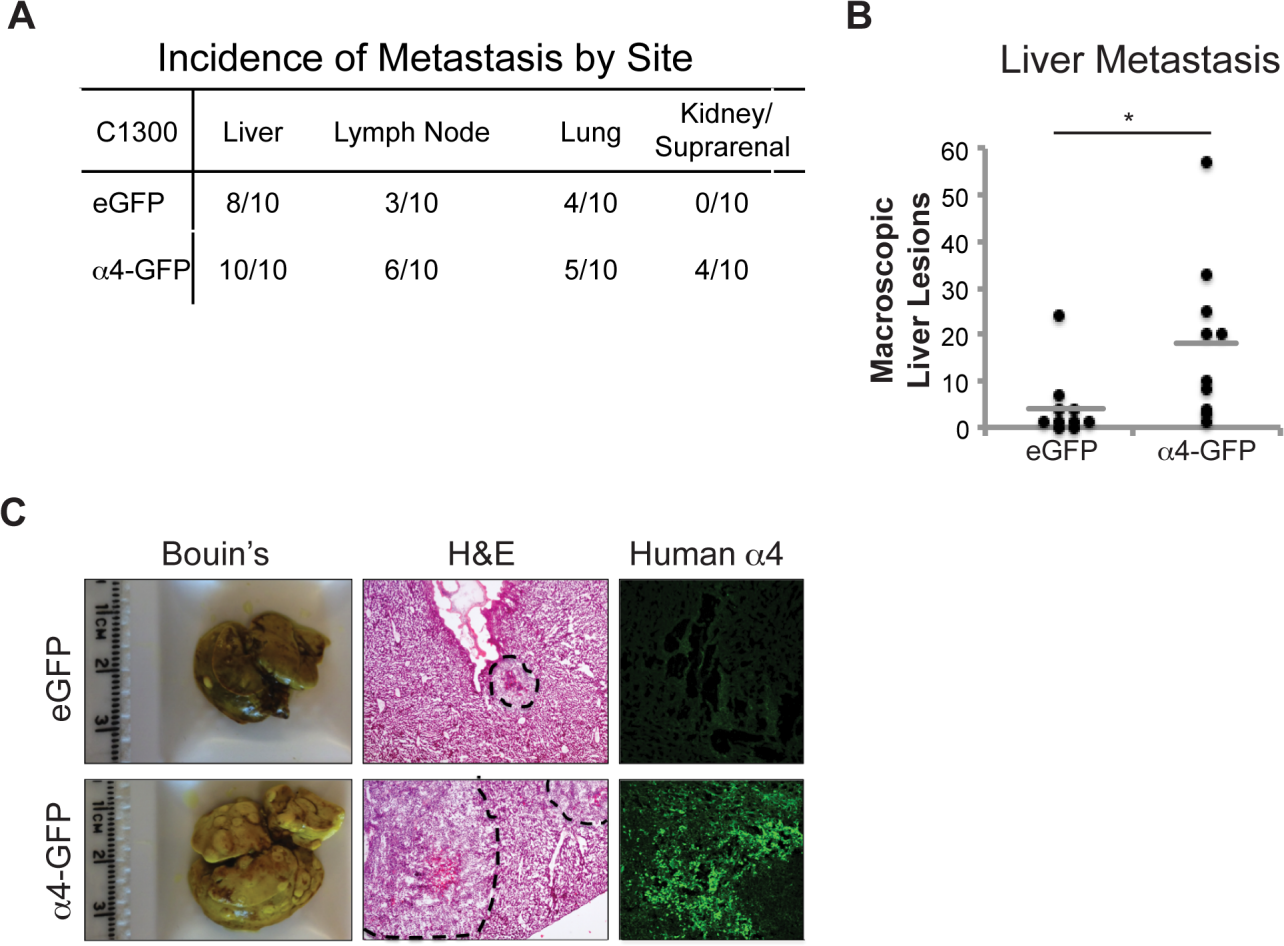
Integrin α4 promotes NB metastasis. (A) C1300 egfp or α4-gfp cells were injected into the tail vein of 8-week-old A/J mice. After 18–24 days, tissues were harvested and examined for macroscopic lesions. (B) The number of macroscopic metastatic liver lesions was assessed by counting after Bouin’s staining (n = 10, p<0.05). Gray lines denote the means of each group. (C) Images of Bouin’s, hematoxylin and eosin, or human integrin α4 stained tissues.

A principal role of integrin α4 is in leukocyte trafficking [9]. To evaluate the effect of integrin α4 expression directly on NB colonization of distant tissues, we characterized the capacity of tumor cells to form metastases as a function of α4 expression. This model reproduced in mice a common clinical vignette in patients; metastases including lymph nodes and liver (Fig 5A). The probability of lymph node metastases was slightly elevated in tumors expressing α4; moreover, the expression of this integrin was associated with metastases detected in kidney/adrenal regions that were not observed in tumors lacking α4. While there was no significant difference in the overall number of mice presenting with hepatic lesions, the number of individual lesions counted on the livers of mice injected was significantly (four fold) higher (Fig 5B and 5C). Together, the results suggest that the expression of α4 significantly promotes metastasis.

We anticipated that the expression of integrin α4 might increase metastasis simply as a byproduct of increased tumor cell enlodgement in the liver capillaries. Examination of the tumor cells present at 24, 48 and 72 hours failed to show a significant difference in the number of tumor cells lodging as a function of α4 integrin expression (S3 Fig). Thus, events that occur following cell arrest may account for the success of the α4-expressing NB at colonization of the liver.

### The α4 cytoplasmic tail is dispensable for adhesion and migration *in vitro*

**Fig 6:**
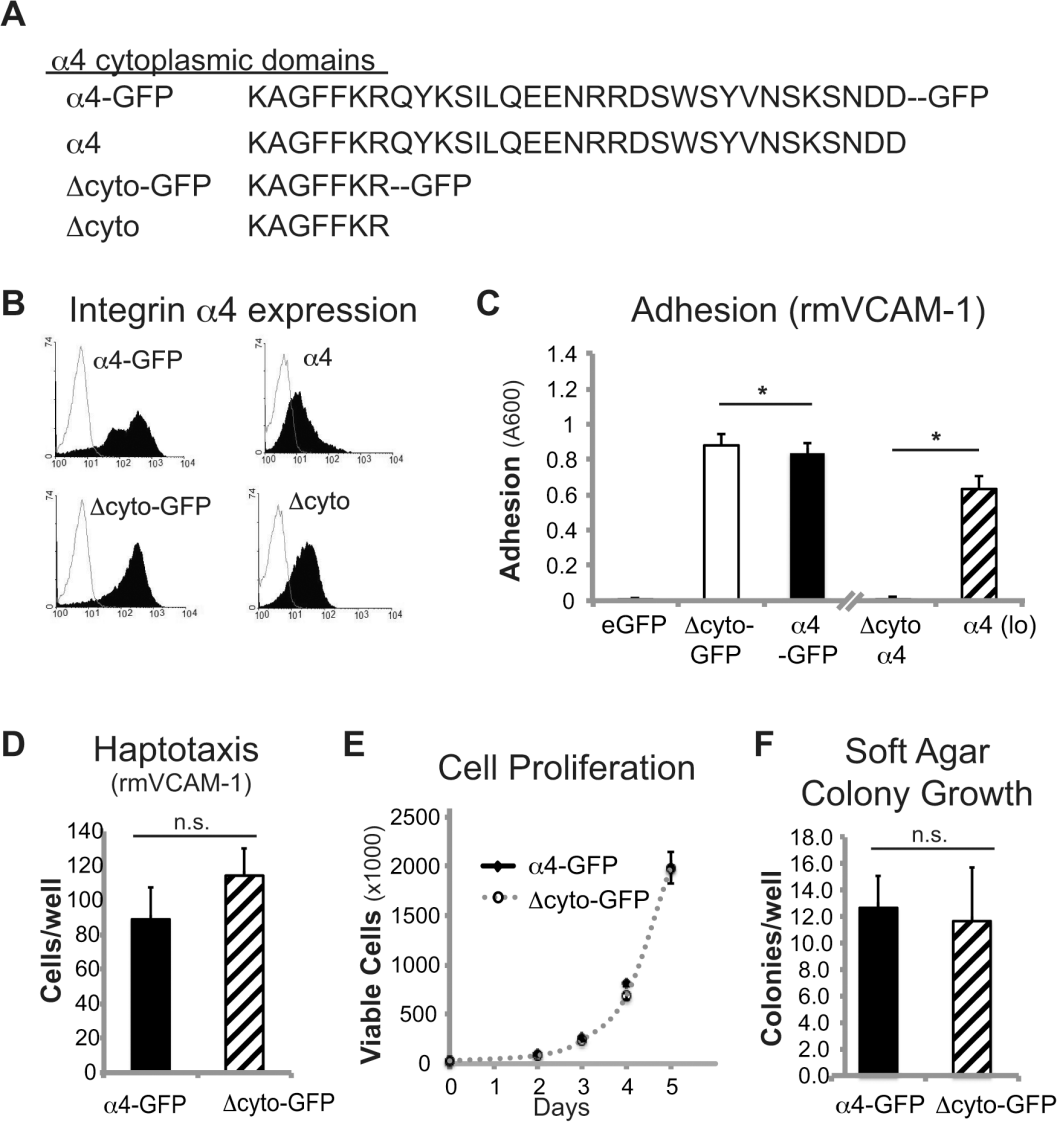
The α4 cytoplasmic tail is dispensible for NB adhesion and migration *in vitro*. (A) Sequences of full-length and truncated α4 cytoplasmic domains. (B) Flow cytometry analysis of C1300 cells stably expressing full-length or truncated α4 and α4-GFP fusion protein (α4 antibody; P1H4) (shaded peaks) compared to secondary only control (open peaks). C1300 cell adhesion (C) and haptotaxis (D) to 2 ug/ml rmVCAM-1. (C) GFP-fusion restores C1300 cell adhesion to wildtype levels (Δcyto vs. Δcyto-GFP, p<0.01). (E) C1300 cell proliferation over 5 days. (F) C1300 colony formation in soft agar after 4 days. Values for proliferation are means ± s.d. (n = 3) of a representative experiment (of two). Values for the soft agar growth assay are the means ± s.d. (n = 6) of a representative experiment.

To begin to address the mechanisms by which α4-expressing cells might show increased metastasis, we evaluated the requirement for the α4 integrin cytoplasmic domain (‘tail’). Integrin tails are essential for a number of integrin-mediated effects [31,32]. C1300 cells stably expressing truncated α4 with a c-terminal GFP tag (α4 Δcyto-GFP) (Fig 6A and 6B) attached to α4 substrates similar to cells expressing the wild-type integrin (Fig 6C). On the surface, the result was somewhat surprising, as it appeared contrast with our prior studies in *MYCN*-amplified NB8 cells, where the cytoplasmic domain of this integrin was found to be critical for mediating cell migration [16]. Nonetheless, it was previously shown that the presence of a surrogate cytoplasmic domain could rescue some α4 integrin functions [33], and in this case, the surrogate tail was the GFP tag.

In fact, while our tagged α4 truncation was able to mediate strong adhesion and migration (Fig 6C and 6D), these were both compromised in cells expressing truncated α4 lacking the GFP tag fusion (α4 Δcyto), which terminate in a simple KAGFFKR sequence (Fig 6C and data not shown). Similar results were obtained for adhesion and migration of human NB with or without amplified MYCN although migration was somewhat compromised in the MYCN amplified NB8 cells (S2B and S2C Fig; S4 Fig), consistent with prior observations of MYCN-mediated downregulation of integrin function [21]. In our studies, the expression of truncated α4 (α4 Δcyto-GFP) had no effect on cell proliferation (Fig 6E) or clonogenic growth (Fig 6F). Thus, we observed no dominant effect associated with expression of the tagged integrin mutant.

### Integrin α4 adhesive function and cytoplasmic tail are critical for α4 integrin-enhanced metastasis

**Fig 7:**
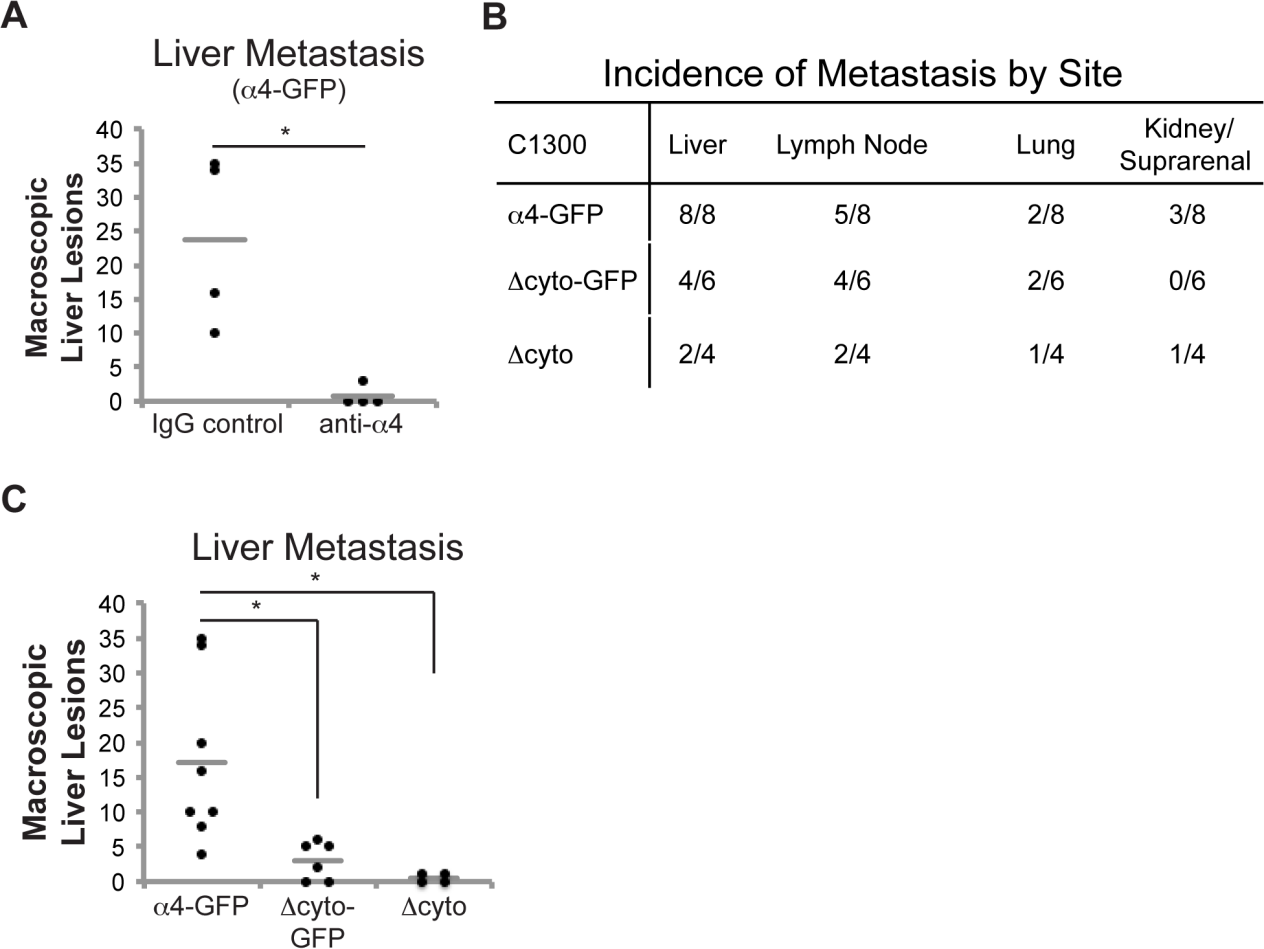
The cytoplasmic tail is important for α4-mediated NB metastasis. (A) Number of metastatic liver lesions in mice receiving tail vein injection of C1300 α4-gfp cells pre-treated with 10 ug/ml anti-α4 antibody (P1H4) or control IgG. Tissues were harvested and examined for metastases after 21 days (n = 4, p<0.05). 1 x 10^**6**^ C1300 full-length or truncated α4 cells were injected into the tail vein of 8-week-old A/J mice (n = 8 α4-GFP, 6 Δcyto-GFP, 4 Δcyto). Tissues were harvested after 21 days. (B) The number of macroscopic metastatic lesions in tissues harvested. (C) Number of macroscopic liver lesions (p<0.01). Gray lines denote the mean of each group (A, C).

To test whether the matrix adhesive function of the integrin tail was required for metastasis, C1300 α4-GFP cells were pre-incubated with a function-blocking antibody to integrin α4 prior to tail vein injection. Blockade of integrin adhesive function resulted in dramatically decreased metastasis (Fig 7A) implying that integrin adhesive function was required. As a complementary approach, we next tested metastasis formation in cells in which integrin function was compromised by deletion of the α4 cytoplasmic domain, or rescued by expression of the GFP tag. The loss of adhesive function in the α4 truncation (α4 Δcyto) was associated with decreased metastasis, as expected (Fig 7B and 7C). Unexpectedly, the α4 Δcyto-GFP cells, in which these functions were reconstituted, were also deficient in metastasis formation (Fig 7C). The results raise the interesting possibility that other post-ligand binding events modulated by the alpha integrin tail may influence the eventual success of the tumor.

## Discussion

Given the role of integrin α4 in the arrest, recruitment and extravasation of leukocytes [9,34], it is perhaps not surprising that its expression might be associated with increased tumor dissemination. However, there is limited data to support this concept, and in fact the amplification of this integrin is not commonly observed in the TCGA database. Except for a small number of uterine, ovarian and prostate tumors, the principal modification of the integrin appears to be mutation. Nonetheless, the role for α4 as a potentiator of tumor cell metastasis is supported by studies in select cancer models where α4 promotes metastasis [35,36]. The reason for this apparent paradox is not clear, but possibly the integrin causes conflicts in cells that do not normally express it.

However, the observation may provide one explanation for the selective expression in *MYCN* ^low^ tumor cells, since expression of integrins without appropriate ligands may promote apoptosis in *MYCN* amplified cells [21,22]. Nonetheless, like *MYCN*, the expression of integrin α4 is shown to be a potential indicator of poor prognosis, and might thus be useful as a target, or a simple diagnostic when choices between more and less aggressive treatment are made.

The mechanism by which integrin α4 promotes metastasis is dependent upon its adhesive function, and provides strong support for the idea that leukocyte mimicry and extravasation are critical factors permitting this integrin to impact neuroblastoma malignancy. Nonetheless, it appears that adhesive function was insufficient, since truncation mutants that were adherent and migrated were nonetheless only slightly more capable of dissemination than completely disabled integrins. The results suggest that the cytoplasmic domain of integrin α4 was critical for tumor metastasis. The reasons for this are not clear, but it is interesting that among the 182 mutations documented in the TCGA database for integrin α4, only 2 fall in the cytoplasmic domain, and both are conservative changes (D1031N in TCGA-ER-A42K, and F1004L in TCGA-BS-A0UF). Overall, we noted no differences in cell growth in two and three dimensional culture systems, or primary tumors, but only observed differences in metastases. The data support the notion that the membrane proximal sequence (KAGFFKR), which is commonly used for deletions [33,37] has significant mobility in the membrane [33]. The results suggest that chimeras, tagged constructs or point mutants be implemented as preferred probes of integrin function [38].

Besides its migratory effects, α4 integrins have roles in cell survival [39]. For example, α4 can activate critical pathways downstream of integrins, including Src, FAK, Akt and ERK [16]. We found no significant differences in any of these mediators following attachment to α4-specific ligands (SAY and DGS, unpublished observations). It is not yet clear if these signaling patterns are representative of 2D, as well as 3D growth, since different signaling patterns and requirements govern 2D versus 3D cell migration and survival [40].

Neuroblastoma prognosis frequently depends upon ploidy, loss of heterozygosity, pathology, and MYCN expression. Although MYCN amplification is likely the strongest predictor of disease progression, α4 may serve as a novel marker for patients lacking this primary indicator. Notably, the cell lines used in this study had low or no amplification of MYCN, directly supporting these clinical observations. Our finding that integrin α4 expression in patient tumor samples was associated with poorer prognosis in MYCN-negative tumors (including lower grade tumors) provides an additional potential tool for the group of tumors with the most ambiguous prognosis.

## Supporting Information

S1 Fig NB5 integrin expression profile. (A) Flow cytometry analysis of integrin expression on NB5 parental cells or cells stably expressing eGFP or full-length α4-GFP fusion protein.
(PDF)

S2 Fig Integrin α4 promotes adhesion and migration the NB8 model. (A) Flow cytometry analysis of integrin α4 expression (shaded peaks) in human NB8 parental cells or in cells sorted for the α4 negative population (SAN) and stably reconstituted with GFP, α4-GFP, or Δcyto-GFP. Geometric mean intensity of α4 positive cells is shown in parentheses. Open peaks represent the secondary only control. Adhesion (B) and haptotaxis (C) of NB8 cells to 5 ug/ml GST-CS1 FN.
(PDF)

S3 Fig Integrin α4 expression does not affect C1300 cellular arrest. (A) C1300 eGFP or α4-GFP cells were stained with CellTracker Red CMPTX and injected into the tail vein of A/J mice. Tissues were harvested 24, 48, and 72 hours after injection and cells arrested in the liver were visualized using the OV-100 imaging system. (B) Quantification of cellular arrest (area of fluorescence) in the liver (left) using ImageJ.
(PDF)

S4 Fig The α4 cytoplasmic tail is dispensible for human NB cell adhesion and migration *in vitro*. (A) Flow cytometry analysis of NB5 cells stably expressing full-length or truncated α4-GFP fusion protein (α4 antibody; P1H4). Adhesion of NB5 α4-GFP and Δcyto-GFP cells to 5 ug/ml GST-CS1 FN after 30 minutes (p<0.01). (C) Scratch wound healing of NB5 α4-GFP or α4 Δcyto-GFP cells seeded on 5 ug/ml pFN or CS1.
(PDF)

S1 Supplemental Materials and Methods (DOCX)
