# Supplementary material for: Integrin α4 Enhances Metastasis and May Be Associated with Poor Prognosis in MYCNlow Neuroblastoma
Source: PLoS One. 2015 May 14;10(5):e0120815. doi: 10.1371/journal.pone.0120815 (PMC4431816; doi:10.1371/journal.pone.0120815)
Supplement: S1 Fig — (A) Flow cytometry analysis of integrin expression on NB5 parental cells or cells stably expressing eGFP or full-length α4-GFP fusion protein. (PDF) [file pone.0120815.s001.pdf]

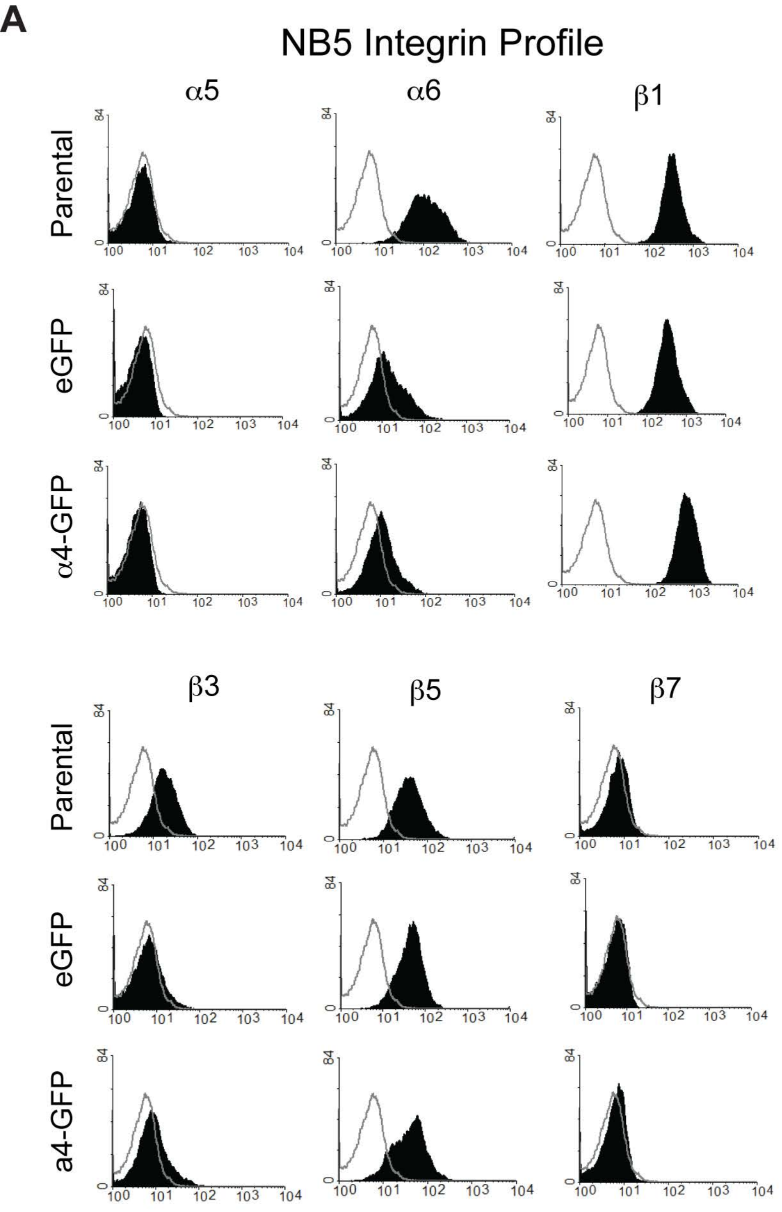

Figure S1. NB5 integrin expression profile. (A) Flow cytometry analysis of integrin expression on NB5 parental cells or cells stably expressing eGFP or full-length α4-GFP fusion protein.
