# Supplementary material for: Integrin α4 Enhances Metastasis and May Be Associated with Poor Prognosis in MYCNlow Neuroblastoma
Source: PLoS One. 2015 May 14;10(5):e0120815. doi: 10.1371/journal.pone.0120815 (PMC4431816; doi:10.1371/journal.pone.0120815)
Supplement: S2 Fig — (A) Flow cytometry analysis of integrin α4 expression (shaded peaks) in human NB8 parental cells or in cells sorted for the α4 negative population (SAN) and stably reconstituted with GFP, α4-GFP, or Δcyto-GFP. Geometric mean intensity of α4 positive cells is shown in parentheses. Open peaks represent the secondary only control. Adhesion (B) and haptotaxis (C) of NB8 cells to 5 ug/ml GST-CS1 FN. (PDF) [file pone.0120815.s002.pdf]

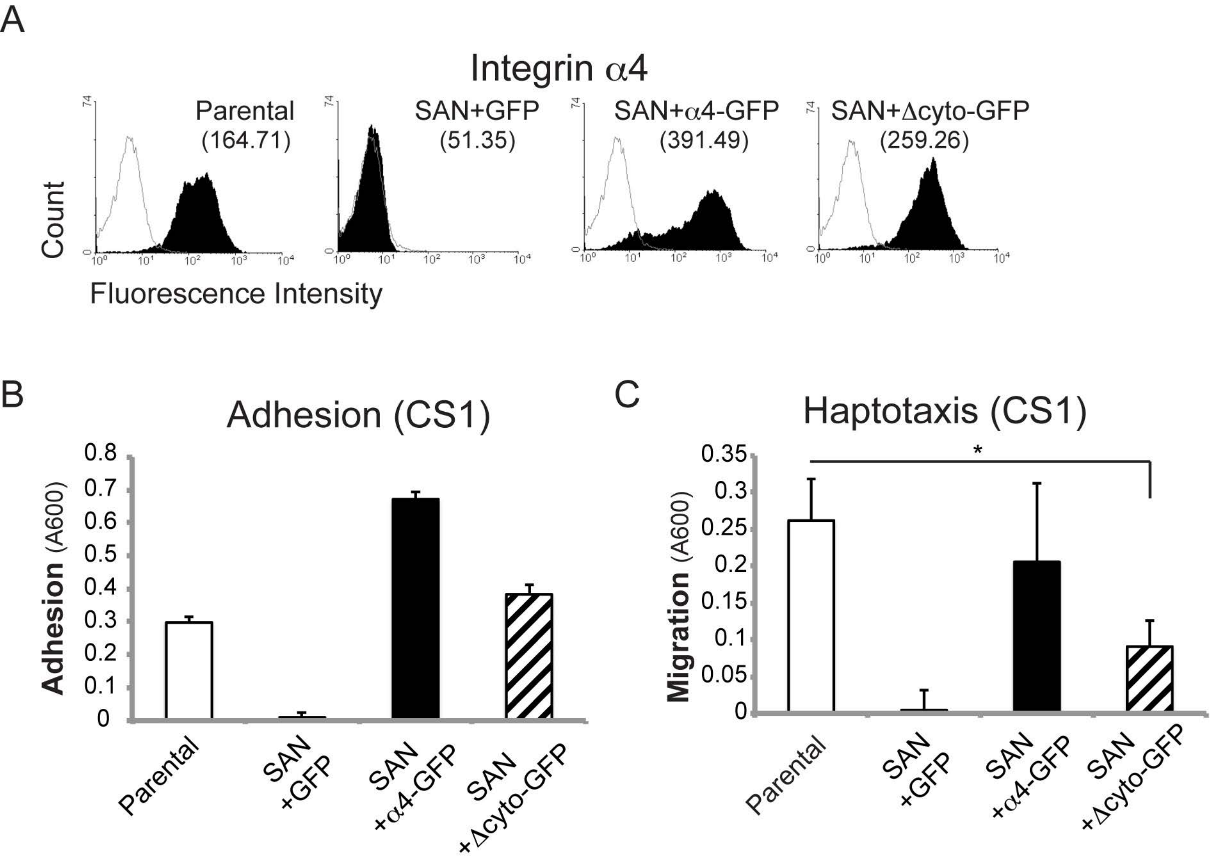

Figure S2. Integrin α4 promotes adhesion and migration in the NB8 model. (A) Flow cytometry analysis of integrin α4 expression (shaded peaks) in human NB8 parental cells or in cells sorted for the α4 negative population (SAN) and stably reconstituted with GFP, α4-GFP, or Δcyto-GFP. Geometric mean intensity of α4 positive cells is shown in parentheses. Open peaks represent the secondary only control. Adhesion (B) and haptotaxis (C) of NB8 cells to 5 ug/ml GST-CS1 FN.
