# Supplementary material for: Integrin α4 Enhances Metastasis and May Be Associated with Poor Prognosis in MYCNlow Neuroblastoma
Source: PLoS One. 2015 May 14;10(5):e0120815. doi: 10.1371/journal.pone.0120815 (PMC4431816; doi:10.1371/journal.pone.0120815)
Supplement: S3 Fig — (A) C1300 eGFP or α4-GFP cells were stained with CellTracker Red CMPTX and injected into the tail vein of A/J mice. Tissues were harvested 24, 48, and 72 hours after injection and cells arrested in the liver were visualized using the OV-100 imaging system. (B) Quantification of cellular arrest (area of fluorescence) in the liver (left) using ImageJ. (PDF) [file pone.0120815.s003.pdf]

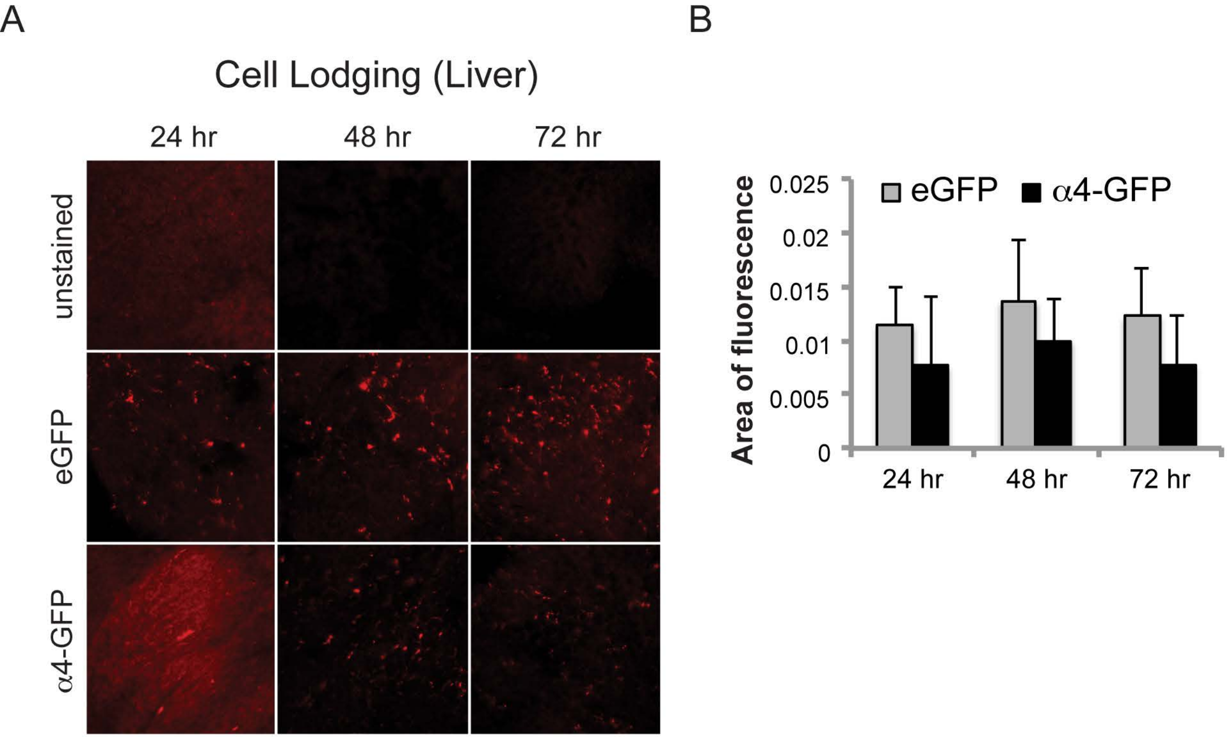

Figure S3. Integrin α4 expression does not affect C1300 cellular arrest. (A) C1300 eGFP or α4-GFP cells were stained with CellTracker Red and injected into the tail vein of A/J mice. Tissues were harvested 24, 48, and 72 hours after injection and cells arrested in the liver were visualized using the OV-100 imaging system. (B) Quantification of cellular arrest (area of fluorescence) in the liver (left) using ImageJ.
