# Supplementary material for: Integrin α4 Enhances Metastasis and May Be Associated with Poor Prognosis in MYCNlow Neuroblastoma
Source: PLoS One. 2015 May 14;10(5):e0120815. doi: 10.1371/journal.pone.0120815 (PMC4431816; doi:10.1371/journal.pone.0120815)
Supplement: S4 Fig — (A) Flow cytometry analysis of NB5 cells stably expressing full-length or truncated α4-GFP fusion protein (α4 antibody; P1H4). Adhesion of NB5 α4-GFP and Δcyto-GFP cells to 5 ug/ml GST-CS1 FN after 30 minutes (p<0.01). (C) Scratch wound healing of NB5 α4-GFP or α4 Δcyto-GFP cells seeded on 5 ug/ml pFN or CS1. (PDF) [file pone.0120815.s004.pdf]

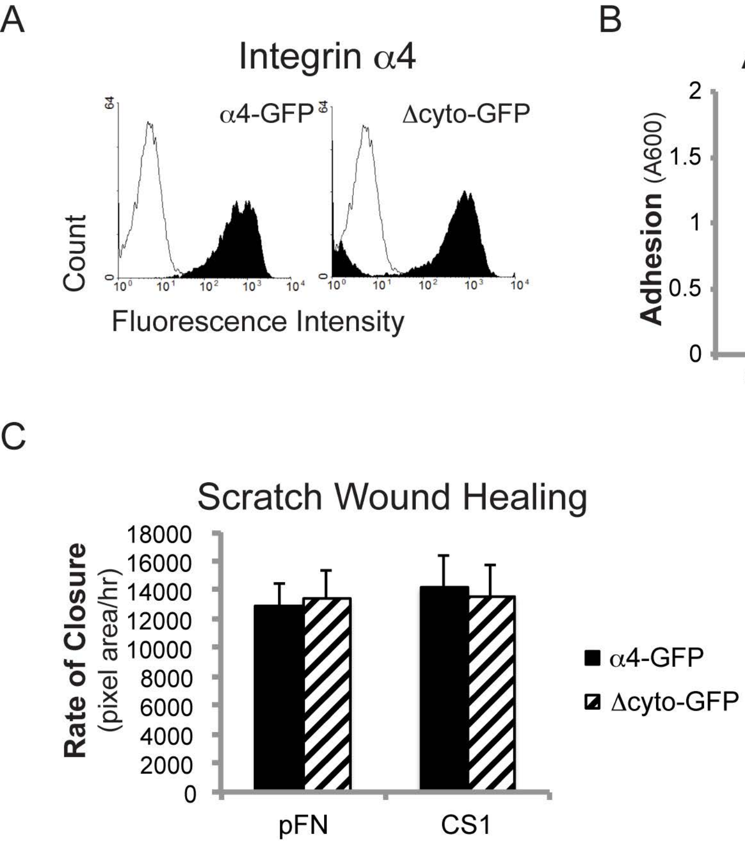

Figure S4. The α4 cytoplasmic tail is dispensible for human NB cell adhesion and migration in vitro. (A) Flow cytometry analysis of NB5 cells stably expressing full-length or truncated α4-GFP fusion protein (α4 antibody; P1H4). Adhesion of NB5 α4-GFP and Δcyto-GFP cells to 5 ug/ml GST-CS1 FN after 30 minutes ($p<0.01$). (C) Scratch wound healing of NB5 α4-GFP or α4 Δcyto-GFP cells seeded on 5 ug/ml pFN or CS1.
