# Supplementary material for: Integrin α4 Enhances Metastasis and May Be Associated with Poor Prognosis in MYCNlow Neuroblastoma
Source: PLoS One. 2015 May 14;10(5):e0120815. doi: 10.1371/journal.pone.0120815 (PMC4431816; doi:10.1371/journal.pone.0120815)
Supplement: S1 Supplemental Materials and Methods — (DOCX) [file pone.0120815.s005.docx]

**Supplemental Materials and Methods**

The following additional protocols and reagents were used to generate the supporting figures presented in this study.

**Antibodies and reagents.** Mouse anti-human integrin α1 (FB12), α2 (P1E6), α5, and α6 antibodies were from Chemicon International. Anti-integrin β7 (FIB504) was from BD. All other antibodies were from Millipore.

**Cell lines.** Human NB8 neuroblastoma cells were cultured under the same conditions and the NB5 and C1300 cells.

***In vivo* cell arrest.** To optimize detection, C1300 eGFP or α4-GFP cells were labeled with CellTracker Red CMPTX (Molecular Probes) according to the manufacturer’s instructions. 1 x 10^6^ cells were injected into the tail vein of A/J mice. Tissues were harvested 24, 48, and 72 hours after injection. Livers were gently minced with a razor blade and flattened between slides. Cells arrested in the liver were visualized using the OV-100 imaging system. Livers from mice injected with unstained cells were used as a control. Quantification of cellular arrest (area of fluorescence) in the liver was performed using the thresholding and measure functions in ImageJ.
